# Molecular Basis of the Versatile Regulatory Mechanism of HtrA-Type Protease AlgW from Pseudomonas aeruginosa

**DOI:** 10.1128/mBio.03299-20

**Published:** 2021-02-23

**Authors:** Tao Li, Yingjie Song, Liming Luo, Ninglin Zhao, Lihui He, Mei Kang, Changcheng Li, Yibo Zhu, Yalin Shen, Chang Zhao, Jing Yang, Qin Huang, Xingyu Mou, Zhiyong Zong, Jinliang Yang, Hong Tang, Yongxing He, Rui Bao

**Affiliations:** a Center of Infectious Diseases, State Key Laboratory of Biotherapy, West China Hospital, Sichuan University and Collaborative Innovation Center, Chengdu, China; b Department of Cancer Biotherapy Center, The Third Affiliated Hospital of Kunming Medical University (Tumor Hospital of Yunnan Province), Kunming, China; c School of Life Sciences, Lanzhou University, Lanzhou, China; d Department of Laboratory Medicine, West China Hospital, Sichuan University, Chengdu, China; University of Pittsburgh School of Medicine

**Keywords:** *Pseudomonas aeruginosa*, crystal structure, AlgW, HtrA, regulated intramembrane proteolysis, mucoid phenotype, alginate

## Abstract

AlgW, a membrane-bound periplasmic serine protease belonging to the HtrA protein family, is a key regulator of the regulated intramembrane proteolysis (RIP) pathway and is responsible for transmitting the envelope stress signals in Pseudomonas aeruginosa. The AlgW PDZ domain senses and binds the C-terminal of mis-localized outer membrane proteins (OMPs) or periplasmic protein MucE, leading to catalytic activation of the protease domain. While AlgW is functionally well studied, its exact activation mechanism remains to be elucidated. Here, we show that AlgW is a novel HtrA protease that can be biochemically activated by both peptide and lipid signals. Compared with the corresponding homologue DegS in Escherichia coli, AlgW exhibits a distinct substrate specificity and regulation mechanism. Structural, biochemical, and mutagenic analyses revealed that, by specifically binding to the C-terminal decapeptide of MucE, AlgW could adopt more relaxed conformation and obtain higher activity than with tripeptide activation. We also investigated the regulatory mechanism of the L_A_ loop in AlgW and proved that the unique structural feature of this region was responsible for the distinct enzymatic property of AlgW. These results demonstrate the unique and diverse activation mechanism of AlgW, which P. aeruginosa may utilize to enhance its adaptability to environmental stress.

## INTRODUCTION

Regulated intramembrane proteolysis (RIP) is a fundamentally conserved mechanism for transmembrane signal transduction in prokaryotes and eukaryotes (1, 2). A typical RIP process is characterized by sequential cleavage of a membrane-spanning precursor that modulates downstream signaling cascades in which a membrane-bound site-1 protease (S1P) initiates proteolytic action on the external side of the precursor protein, and a membrane-embedded site-2 protease (S2P) is responsible for subsequent cleavage on its transmembrane segment (3). Pseudomonas aeruginosa, a Gram-negative opportunistic pathogenic bacterium, possesses a functional MucA-RIP system composed of precursor MucA and its periplasmic partners MucB, S1P AlgW, S2P MucP, and cytoplasmic ClpXP protease (4). This signaling cascade plays crucial roles in response to extracellular stresses and governs mucoid conversion, flagellum synthesis, cell envelope homeostasis, and virulence genes expression (5).

Amino acid sequence analysis showed that AlgW was homologous to Escherichia coli DegS and belonged to the HtrA-family (6). HtrA protease is characterized by its trypsin-like catalytic domain and regulatory PDZ domain; the catalytic triad was surrounded by several loops (L_1_, L_2_, L_3_, L_D_, L_A_) but, usually, in an inactive mode, and could be activated by sensing a specific molecular stimulus (7). PDZ domains serve as protein sensors that allow RIP systems to respond to the accumulated stress signals in periplasm, including mis-localized outer membrane protein OMPs and exfoliated lipids (8, 9). Previous structural studies revealed that the PDZ domain of DegS functions as an inhibitory element by interacting with L_3_ of the protease domain, whereas PDZ provides a binding site for the YxF signature of activating peptides (10). Compared with DegS, AlgW displayed different recognition specificities toward the OMP C-terminal motif, and its PDZ domain has indispensable roles in both repressing and activating proteolytic activity (6, 11). Such differences raise questions as to what is the structural basis underlying the distinct properties of AlgW. Does there exist any other specific features of AlgW, and what impacts do those features have on MucA-RIP system of P. aeruginosa?

In this study, we found that both MucA and MucB were subjected to AlgW cleavage. Notably, in addition to providing the molecular basis of the unique peptide activation mechanism, we demonstrated that the lipid molecule could stimulate AlgW activity as well, indicating a dual signal amplification mechanism of AlgW in the MucA-RIP system. Furthermore, the combination of peptide and lipid resulted in a synergistic effect. In order to gain insights into the structural features of AlgW, we determined a set of crystal structures of peptide-bound AlgW representing different activation states. Structural analysis showed that the significant conformation changes of AlgW, especially the PDZ domain and L_A_ loop, were associated with the peptide effector binding. Mutagenesis and biochemical analysis further evidenced that the unique features of AlgW are important regulation mechanisms and are essential for the biological functions of the MucA-RIP system. These findings provide a basis to understand the distinct properties of AlgW, which may reflect a rapid action mechanism of envelope stress responses in P. aeruginosa.

## RESULTS

### Cleavage of MucA and MucB by AlgW in response to the dual stimuli.

**FIG 1:**
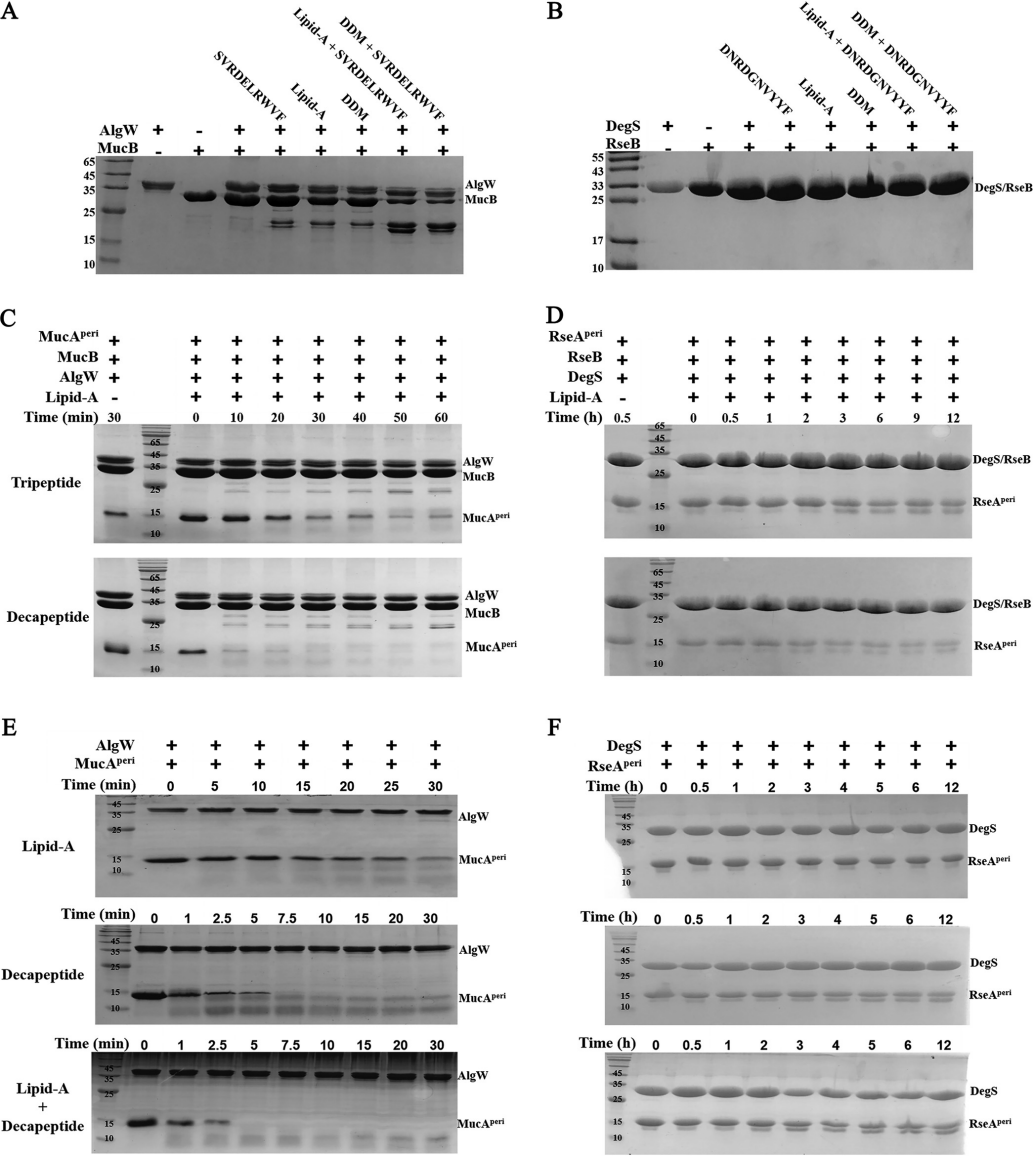
Degradation of MucA and MucB by AlgW in response to the peptides and lipid signals. (A and B) MucB/RseB (130 μM) was cleaved by AlgW/DegS (25 μM) in the presence of different agonist combinations, including lipid-A (100 μM), lipid-like detergent DDM (400 μM), decapeptide SVRDELRWVF (100 μM), and decapeptide combined with lipid-A or DDM. (C and D) MucB/RseB-protected MucA^peri^/RseA^peri^ degradation by AlgW/DegS in the presence of different lengths of the activating peptides in a time-dependent manner. MucA^peri^ (125 μM), AlgW (25 μM), MucB (130 μM), activation peptides (tripeptide WVF or decapeptide SVRDELRWVF, 100 μM), and lipid-A (100 μM) were incubated at 37°C in a time-dependent manner in buffer (25 mM Tris-HCl pH 7.5, 150 mM NaCl). Similarly, RseA^peri^ (125 μM) was cleaved by DegS (25 μM) in the presences of RseB (130 μM), activation peptides (tripeptide YYF or decapeptide DNRDGNVYYF, 100 μM), and lipid-A (100 μM) at 37°C in a time-dependent manner in a phosphate buffer containing 50 mM NaHPO_4_ (pH 7.5), 200 mM NaCl, 10% glycerol, and 4 mM EDTA. (E and F) MucA^peri^/RseA^peri^ was cleaved by AlgW/DegS in the presence of different agonist combinations in a time-dependent manner. MucA^peri^ (125 μM), AlgW (25 μM), activation decapeptide SVRDELRWVF (100 μM), and lipid-A (100 μM) were incubated at 37°C in a time-dependent manner in buffer. Similarly, RseA^peri^ (125 μM) degradation by DegS (25 μM) took place in the presence of RseB (130 μM), activation decapeptide DNRDGNVYYF (100 μM), and lipid-A (100 μM) at 37°C for a time-dependent manner in a phosphate buffer.

The MucA-RIP system is homologous to the E. coli RseA-RIP system (12). Correspondingly, AlgW, MucA, and MucB are analogous to DegS, RseA, and RseB, respectively. MucB/RseB can form a heterodimeric complex with MucA/RseA to suppress the S1P (AlgW/DegS) cleavage, while the accumulated periplasmic lipopolysaccharide (LPS) would prevent the complex formation by directly interacting with the lipid-binding domains of MucB/RseB (9, 13). However, unlike DegS, which only acts on RseA but not RseB, AlgW in the MucA-RIP system can recognize and degrade both MucA and MucB (13). In order to investigate the cleavage specificity of AlgW, we mixed MucB with the soluble AlgW (residues 30 to 389, without the N-terminal transmembrane segments), supplying decapeptide SVRDELRWVF (MucE C-terminal tail) as the activator, and degraded MucB bands were observed after 30 min of incubation (Fig. 1A). In contrast, no proteolysis was noticed in the DegS-RseB group when it was supplied with decapeptide DNRDGNVYYF (14) (Fig. 1B). In our previous work (9, 13), when the MucA/MucB complex, AlgW, peptide agonist (for AlgW activation), and lipid/detergent (to antagonize MucA-MucB association [9, 13]) were mixed to reconstitute the proteolysis events of the MucA, we observed that AlgW could be activated by lipid-A (LPS glycolipid moiety) or detergent DDM (*N*-dodecyl-β-d-maltopyranoside) (Fig. 1A). It should be noted that lipid could significantly enhance the decapeptide activation effect on AlgW and hence promoted the cleavage of MucB, implying a synergistic effect between the two types of effector. Collectively, the above-described results suggest that AlgW possesses distinct properties that are likely to contribute to the unique character of the P. aeruginosa MucA-RIP pathway.

In order to compare the effects of lipid molecules on AlgW/DegS during the MucA/RseA proteolysis process and to figure out whether the length of the activating peptide might account for any of the observed differences, we repeated the peptide/lipid-regulated S1P cleavage experiments *in vitro*. The periplasmic domain of MucA (MucA^peri^) and RseA (RseA^peri^) in complex with MucB and RseB, respectively, were subjected to tripeptide or decapeptide activated AlgW/DegS cleavage. Lipid-A was added in each series of experiments, and the results were analyzed in the time scale of minutes (Fig. 1C and D). The results confirmed that both tripeptide and decapeptide could stimulate AlgW activity, while AlgW supplied with decapeptide exhibited much higher activity than that supplied with tripeptide (Fig. 1C). However, DegS showed no obvious difference between different peptide activation assays (Fig. 1D). This result indicates that AlgW is, in principle, similar to DegS in respect to peptide activation, but it has a different regulation mechanism. Simultaneously, specific cleavage of MucB was observed after extensive AlgW digestion, suggesting that AlgW possesses broader substrate specificity than DegS.

Subsequent MucA^peri^/ResA^peri^ degradation experiments confirmed the unique effects of peptide and lipid signals on AlgW (Fig. 1E); in contrast, lipid-A did not display obvious direct or cooperative activation on DegS (Fig. 1F). Additionally, the time-dependent measurements also revealed that peptide had more profound effects than lipid, implying distinct action mechanisms of different types of agonists.

### Investigating the dual signal activation of AlgW using a fluorescence-based quantitative assay.

**FIG 2:**
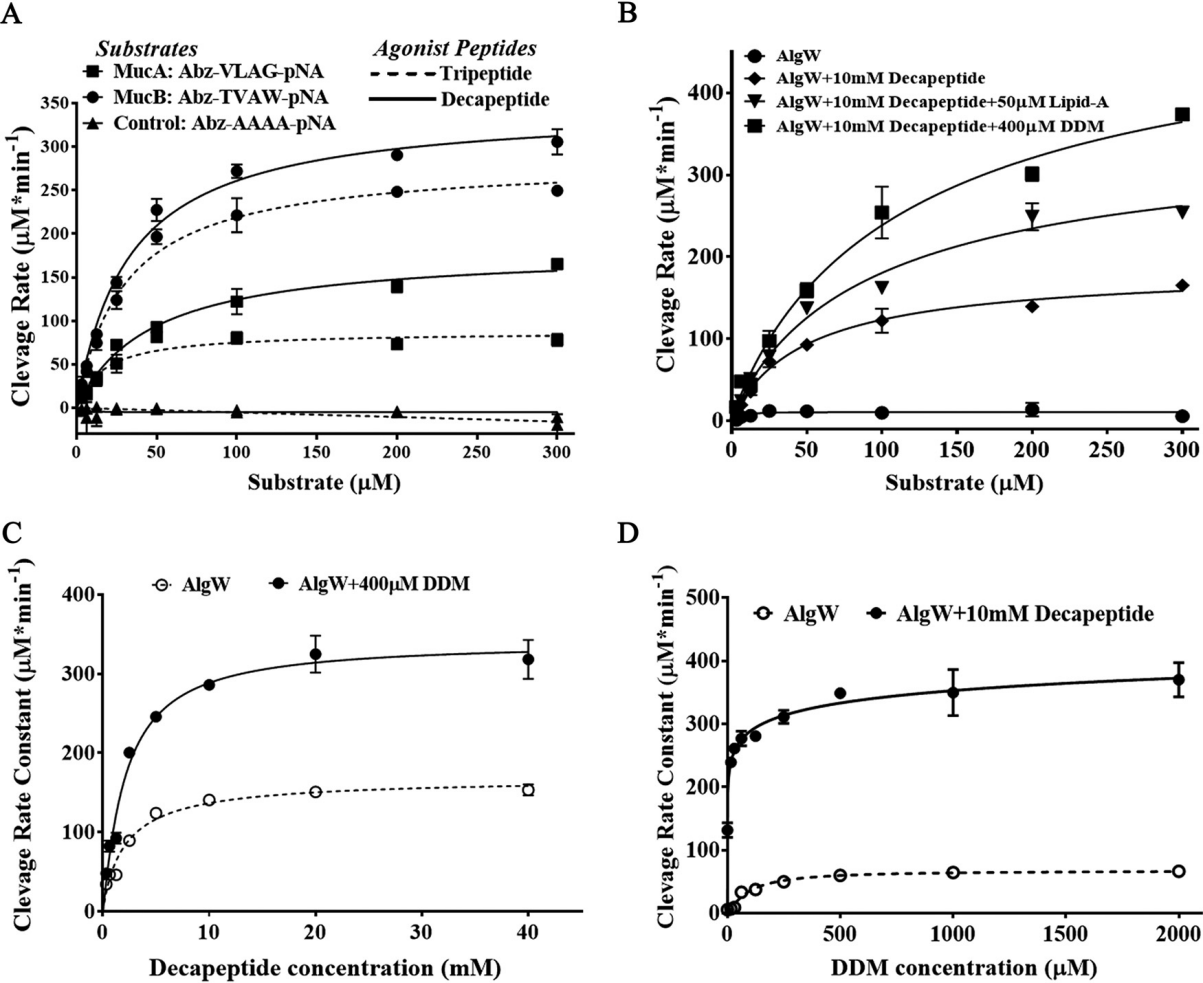
Determination of enzyme apparent kinetic parameters of dual-signal-activated AlgW. (A) Activity assay of tripeptide or decapeptide-activated AlgW using the quenched fluorescent substrates Abz-VLAG-pNA, Abz-TVAW-pNA, and Abz-AAAA-pNA. All assays were placed in a buffer containing 25 mM Tris (pH 7.5) and 150 mM NaCl at 37°C for 1 h by adding 100 μM substrate, 200 nM AlgW, and 10 mM agonist peptides (tripeptide WVF or decapeptide SVRDELRWVF). (B) The apparent Michaelis-Menten parameters of AlgW cleavage quenched fluorescent substrates Abz-VLAG-pNA (100 μM) under the condition of peptide or lipid activation. (C) The activity assay of peptide or DDM activated AlgW using the saturated quenched fluorescent substrates Abz-VLAG-pNA. Under the gradient of peptide or DDM concentration,100 μM substrate and 200 nM AlgW were coincubated with or without 400 μM DDM or 10 mM agonist peptides in a buffer containing 25 mM Tris (pH 7.5) and 150 mM NaCl at 37°C for 1 h. The final assay volume of all experiments was 100 μl, and the appearance of fluorescent products was monitored at 60-s intervals at fluorescence wavelengths of λex = 310 nm and λem = 420 nm. All data points were representative of three independent determinations and were simulated with solid lines through the formula of Y = *V*_max_ · *X*^h/(*K_m_*^h + *X*^h) or *Y* = *V*_max_ · *X*^h/(*K*_half_^h + *X*^h). The *K*_cat_ for the AlgW trimer was calculated by *K*_cat_ = *V*_max_/(*E*_total_), (*E*_total_) = 200/3 nM trimers. Error bars represent the standard deviation.

Based on the identified MucA and MucB cleavage sites for AlgW (6, 13), self-quenching fluorogenic tetrapeptides derived from 134-VLAG-137 of MucA (Abz-VLAG-pNA), 211-TVAW-214 of MucB (Abz-TVAW-pNA), and alanine tetrapeptide (Abz-AAAA-pNA) were synthesized and subjected to real-time measurement of AlgW proteolytic activity in the presence of excessive peptides (10 mM) (Fig. 2A). Compared with the negative-control group (using Abz-AAAA-pNA substrate), both Abz-VLAG-pNA and Abz-TVAW-pNA could be efficiently cleaved by AlgW. We found that AlgW showed an allosteric response to changes in MucB substrate peptide concentrations, with Hill constants of 1.25 for tripeptide activation and 1.26 for decapeptide activation (Fig. 2A, Table S1). However, the binding of MucA substrate peptide to AlgW was only positively cooperative in the presence of tripeptide but exhibited significant allosteric effects upon decapeptide activation (Hill constant near 1), suggesting that the allosteric property of AlgW is linked to a specific activator and substrate (Table S1). Considering that MucA is the major target of AlgW, substrate Abz-VLAG-pNA and decapeptide activator SVRDELRWVF were selected to establish a real-time AlgW assay to investigate the kinetic mechanism.

10.1128/mBio.03299-20.3 TABLE S1 Activity assay of tripeptide- or decapeptide-activated AlgW. Download Table S1, DOCX file, 0.02 MB. Copyright © 2021 Li et al. 2021 Li et al. https://creativecommons.org/licenses/by/4.0/ This content is distributed under the terms of the Creative Commons Attribution 4.0 International license.

Next, we tested the synergistic effect of lipid-A and DDM (Fig. 2B). Consistent with the results in Fig. 1, peptide effector alone and a dual effector combination both exhibited enhancing effects on AlgW, in which the apparent maximum rate of metabolism (*V*_max_) values of the dual effector activation were 1.9- to 2.8-fold higher than that of decapeptide activation (Fig. 2B, Table S2). The catalytic efficiency of different groups was similar (*K*_cat_/*K_m_* = 55 to 65 min^−1 ^μM^−1^) because the apparent *K_m_* values were proportionally associated with the *V*_max_. Consistently, MucA substrate peptide did not exhibit obvious allosteric binding to AlgW when lipid effector was added; therefore, decapeptide-activated AlgW cleavage on Abz-VLAG-pNA could be described by a Michaelis-Menten equation (Table S2). Meanwhile, considering that the low purity and solubility of lipid-A may influence the measurement, we used DDM as a lipid signal substitute in the following quantitative assays.

10.1128/mBio.03299-20.4 TABLE S2 Apparent Michaelis-Menten parameters of AlgW activated by peptides or lipids. Download Table S2, DOCX file, 0.02 MB. Copyright © 2021 Li et al. 2021 Li et al. https://creativecommons.org/licenses/by/4.0/ This content is distributed under the terms of the Creative Commons Attribution 4.0 International license.

In the presence of a fixed amount of AlgW and saturated substrate, we measured the initial rates of AlgW at various concentrations of decapeptide or DDM (Fig. 2C). DDM only exhibited low activation effects but positive cooperativity, where the maximal cleavage activity was about 67.82 ± 3.02 μM min^−1^, with a half-maximal activation concentration of 92.02 ± 12.44 μM and a Hill constant of 1.43 (Table S3). In comparison, decapeptide was more efficient for AlgW activation, which largely increased the cleavage rate up to 168.5 ± 8.27 μM min^−1^ (about 2.7-fold higher than the maximal activated rate by DDM) at high decapeptide concentrations. Meanwhile, the much smaller half-maximal activation concentration (2.12 ± 0.33 μM) indicates tighter interaction of decapeptide to AlgW, and the Hill constant of 0.94 suggests no allosteric effect on decapeptide binding. We also analyzed the synergistic relationship between the two types of activators (Fig. 2C, Table S3); a significant increase of cleavage rates was observed when adding another type of activator in the reaction system, indicating that the peptide and lipid signals have additive effects to each other. At the same time, DDM did not significantly alter the half-maximal activation concentration and Hill constant of decapeptide, implying that peptide and lipid signals have different interaction patterns toward AlgW.

10.1128/mBio.03299-20.5 TABLE S3 Apparent activation parameters of AlgW activated by agonist peptides and DDM. Download Table S3, DOCX file, 0.01 MB. Copyright © 2021 Li et al. 2021 Li et al. https://creativecommons.org/licenses/by/4.0/ This content is distributed under the terms of the Creative Commons Attribution 4.0 International license.

### Crystal structures of AlgW in complex with peptide agonists.

**FIG 3:**
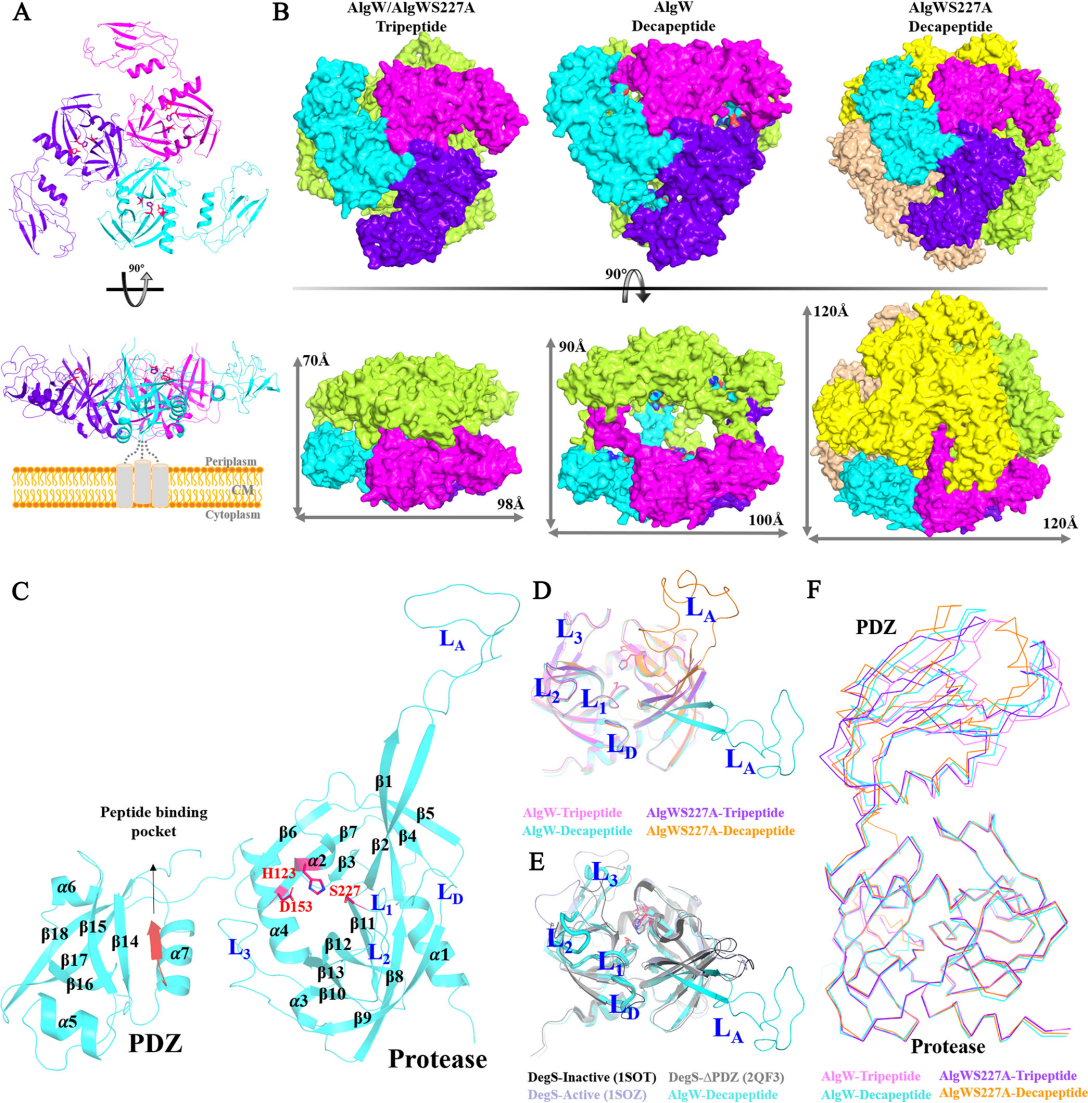
Peptide-bound AlgW structures. (A) Structures and funnel-like trimeric organization of peptide-bound AlgW. Side view of the trimer illustrating the relative orientation of AlgW in the periplasm. (B) The oligomeric structure of AlgW-tripeptide/AlgW-S227A-tripeptide, AlgWS227A-decapeptide, was generated by symmetry operation. Each monomer in all structures was displayed in magenta, blue, and cyan. (C) The overall structures of decapeptide-bound AlgW. The decapeptide bound in AlgW was displayed as red cartoon, and the catalytic triad (H123, D153 and S227) sites surrounded by activation loops (L_1_-L_3_, L_D_ and L_A_) were displayed as red sticks. (D) Structural comparison of the protease domains of peptide-bound AlgW structures. The L_A_ loop in tripeptide-bound AlgW structures cannot be modeled. The joint angle of the L_A_ loop in two sets of decapeptide-bound AlgW structures was calculated using UCSF ChimeraX (http://www.cgl.ucsf.edu/chimerax/index.html), which was 7.65°. (E) Structural comparison of the protease domains of AlgW-decapeptide and DegS structures. L_2_ and L_3_ of AlgW adopt inward conformations compared with those of DegS structures. The catalytic triad sites are displayed as sticks. (F) Structural variations of peptide-bound AlgW structures. The joint angle between the PDZ and the protease domain was defined between the longest inertial vectors of each domain, which was calculated using UCSF ChimeraX. The calculated joint angles in the structures of AlgW-tripeptide, AlgWS227A-tripeptide, AlgW-decapeptide and AlgWS227A-decapeptide were 25.62°, 25.74°, 26.13°, and 26.65°.

In order to elucidate the structural basis of the AlgW reaction mechanism, we prepared the periplasmic soluble domain of AlgW and its inactive mutant AlgWS227A. Protein samples were mixed with two types of peptide activator (tripeptide WVF, decapeptide SVRDELRWVF) prior to crystallization trials, yielding four complex structures, AlgW-tripeptide, AlgWS227A-tripeptide, AlgW-decapeptide, and AlgWS227A-decapeptide, with resolutions from 1.8 to 2.6 Å (Table 1). AlgW-tripeptide and AlgWS227A-tripeptide crystals belong to the P6_3_22 space group. They have only one complex in an asymmetric unit, and their overall structures are similar (the root mean square deviation [RMSD] value was 1.07 Å). The AlgW trimer could be generated by symmetry operation, and similar to most HtrA protease, this funnel-like trimeric organization is mainly mediated by the N-terminal protease domains (Fig. 3A). The trimeric structures are also found in AlgW-decapeptide and AlgWS227A-decapeptide, but they are assembled into higher oligomers (Fig. 3B). In AlgW-decapeptide, each asymmetric unit contains a DegP-like hexamer that is stacked by two interlocked basic trimers arranged in a face-to-face manner (15), whereas the intertrimer contacts are stabilized by the long L_A_ loop extending from the protease domain into the PDZ domain of another trimer. AlgWS227A-decapeptide could even generate a DegQ-like “cage” architecture after symmetry operation (16, 17); the dodecamer of soluble AlgW contains four trimers, with the 12 L_A_ loops protruding toward the interior of the cage (Fig. 3B). L_A_-mediated higher oligomerization is an important regulation mechanism in the HtrA family (18, 19); however, very few close contacts are observed between trimers in AlgW-decapeptide and AlgWS227A-decapeptide structures, indicating that crystal packing may, in fact, dominate the above-described intertrimer associations. In addition, AlgW is an inner membrane-anchored protease; because of the spatial restrictions, it appears to function as a trimer *in vivo* (Fig. 3A and B).

**TABLE 1:** Data collection and structure refinement statistics of peptide-bound AlgW structures

	AlgW-Tripeptide	AlgWS227A-Tripeptide	AlgW-Decapeptide	AlgWS227A-Decapeptide
Protein Data Bank code	7CO2	7CO3	7CO5	7CO7
Space group	P 6_3_ 2 2	P 6_3_ 2 2	P 2_1_ 2_1_ 2_1_	F 2 3
Cell dimensions				
*a*, *b*, *c* (Å)	95.845, 95.845, 118.765	96.614, 96.614, 119.55	95.12, 130.837, 250.364	179.1, 179.1, 179.1
α, β, γ (°)	90, 90, 120	90, 90, 120	90, 90, 90	90, 90, 90
Wavelength (Å)^*a*^	0.97891	0.97930	0.97930	0.97853
R_sym_ (%)^*b*^	0.143 (0.821)	0.097 (0.662)	0.262 (0.618)	0.166 (0.842)
Average I/σ (I)	24.67 (4)	40.1 (5.5)	2.96 (1.34)	22.53 (3.3)
R_meas_	0.145 (0.840)	0.098 (0.675)	0.310 (0.757)	0.169 (0.860)
R_pim_	0.027 (0.176)	0.018 (0.133)	0.162 (0.428)	0.029 (0.178)
Completeness (%)	99.86 (99.58)	99.80 (98.27)	96.1 (94.5)	100 (100)
CC_1/2_	1.05 (0.243)	0.958 (0.915)	0.796 (0.262)	1.00 (0.146)
CC*	1.004 (0.626)	0.989 (0.987)	0.941 (0.644)	1.00 (0.505)
Redundancy	26.5 (21.9)	28.5 (25.2)	3.4 (2.6)	30.3 (23.2)
Refinement				
Resolution (Å)	39.18–2.098 (2.173–2.098)	39.49–1.898 (1.996–1.898)	33.49–2.345 (2.429–2.345)	34.47–2.601 (2.694–2.601)
No. of reflections	19,460 (1,891)	26,613 (2,558)	125,194 (11,425)	14,740 (1,500)
R_work_/R_free_^*c*^	0.2314/0.2530	0.2185/0.2475	0.2639/0.2859	0.1896/0.2267
	(0.3529/0.3701)	(0.3515−0.3623)	(0.3694/0.3522)	(0.3374/0.3439)
No. of atoms	2,378	2,371	15,252	2,575
B-factor from Wilson plot (Å^2^)	34.75	25.71	14.08	58.10
Average B-factor	66.77	60.41	33.31	86.35
Protein	66.32	60.46	33.57	86.35
Ligand/ion	90.40	0	32.80	147.31
Water	71.58	59.83	23.20	83.79
RMSD^*d*^				
Bond lengths (Å)	0.019	0.018	0.024	0.020
Bond angles (°)	2.03	2.15	2.17	2.06
Number of TLS groups	1	1	0	1
Ramachandran plot				
Favored/allowed/ outliers	98.31/1.69/0	96.28/3.72/0	97.88/1.82/0.30	96.69/2.41/0.90

a Values in parentheses are for highest-resolution shell.

b R_sym_ is the unweighted R value on I between symmetry mates.

c R_free_ is calculated analogously for the test reflections, randomly selected and excluded from the refinement.

d RMSD, root mean square deviation.

The overall structure of AlgW consists of 18 β-slices and 7 α-helices (Fig. 3C). The core structure of the AlgW protease domain adopts a typical trypsin fold consisting of two perpendicular β-barrels (β1 to β7, β8 to β13) flanked by two α-helices (α2, α3). The catalytic triad (H123, D153, and S227) sites are located between two β-barrels and are surrounded by activation loops L_1_ to L_3_, L_D_, and L_A_ (named according to DegS nomenclature [20–22]). The majority of the active loops (e.g., L_1_, L_2_, L_3_, and L_D_) except the L_A_ loop are ordered in a uniform conformation in all of the four peptide-bound AlgW structures (Fig. 3D). L_2_ and L_3_ of AlgW adopt inward conformations compared with those of DegS structures, which stabilized the local conformation of catalytic sites (Fig. 3E). The PDZ domain of AlgW has 5 β-strands (β14 to β18) and 3 α-helices (α5, α6, and α7); agonist peptides bind to the groove composed of β14 and α7 and form an antiparallel β-sheet with β14 (Fig. 3C). Unlike the relatively rigid core structure of the protease domain, PDZ domains exhibit conformational plasticity among individual AlgW subunits, resulting in RMSD values of 1.40 to 1.75 Å and different domain arrangements (Fig. 3F).

### Specific peptide recognition and regulation mechanism of AlgW.

**FIG 4:**
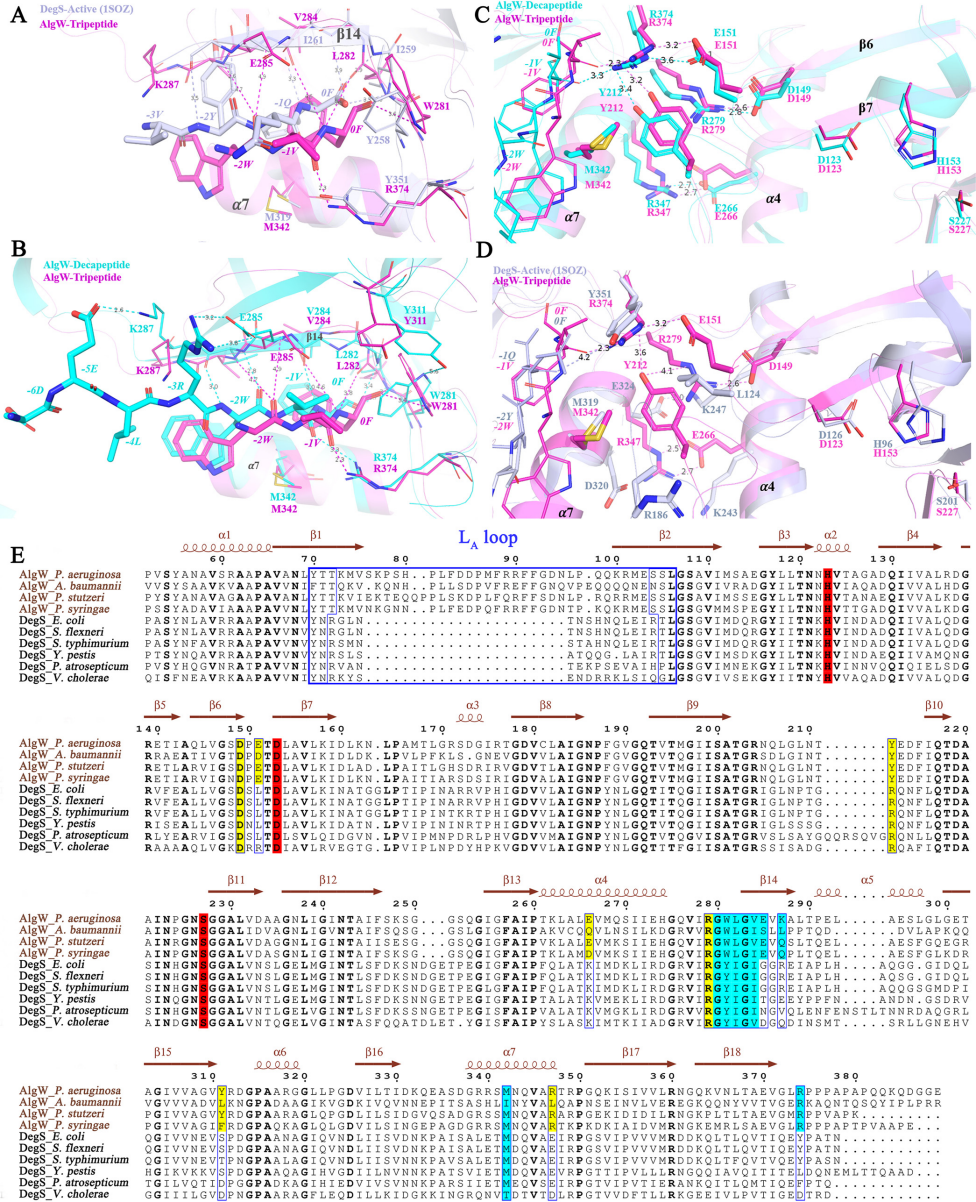
The peptide-binding pocket and domain interactions of AlgW and DegS. (A and B) The interactions of agonist peptides with AlgW/DegS. The key residues in the peptide-binding pockets are displayed as sticks. Peptides are displayed as thick sticks. (C and D) The domain-domain interactions in which the peptide binds to PDZ and mediates the activation of the catalytic triad. The key residues mediating the domain-domain interactions are displayed as sticks. E. Multisequence alignment (generated using Espript 3.0; http://espript.ibcp.fr/ESPript/ESPript/) of AlgW/DegS homologues. The residues in the peptide-binding pocket of AlgW and DegS are shaded in cyan, and the catalytic triad (H123, D153, and S227 in AlgW, H96, and D126 and S201 in DegS) sites are shaded in red. The conserved residues involved in domain-domain interactions are shaded in yellow.

The PDZ domain is well known as a regulatory module recognizing hydrophobic residues at the C terminus of targeting proteins (21, 23). For most PDZ-binding peptides, positions 0 and –2 primarily determine the binding specificities by interacting with the “G-X-G motif” at one end of the α/β groove (24, 25). AlgW and DegS share similar hydrophobic environments to accommodate the large side chains of aromatic residues 0 and –2 (for instance, WVF for AlgW and YQF for DegS) in peptide activators (Fig. 4A) (20). However, there are different compositions and sizes of residues in the corresponding pockets in the PDZ domain of AlgW and DegS, which would account for why AlgW but not DegS allows larger tryptophan residues to fit in (11, 26).

Systematic studies of PDZ-ligand interactions have revealed that positions other than 0 to –2 may also contribute to the peptide-binding specificities (27, 28), Accordingly, AlgW has its second Gly of the “G-X-G” motif replaced by E285, which constrains the size of the –1 residue in peptide ligand (11). Compared with the tripeptide-bound AlgW, decapeptide has more proximal contacts to PDZ, inducing side chain rotation on W281 and thus allowing repositioning of the Y311 (Fig. 4B). Additionally, decapeptide-bound AlgW illustrates specific charge-charge interactions such as –3R to E285 and –5E to K287, which enhances the peptide ligand selectivity (Fig. 4B). The specific peptide-binding features of AlgW were further verified by mutagenesis and activity assay (Table 2). As expected, alanine substitutions on sites W281, L282, and V284 that were involved in the hydrophobic interaction with the C terminus of peptide activator all led to a dramatic decrease in the cleavage rate (Table 2). The increased half-maximum activation concentration in L282A and V284A verified the importance of the hydrophobic pocket which accommodates the 0F. Similarly, E285A and K287A also reduced activity and influenced peptide binding, which confirmed the contribution of the additional charge contacts to peptide-binding specificity. Importantly, mutations on the opposite side of the peptide-binding groove (M342A and R374A) almost abolished the peptide-induced protease activity (Table 2); the explanation is that these sites not only stabilize the anchored peptide but also mediate the domain-domain interactions (Fig. 4B).

**TABLE 2:** Apparent activation parameters of AlgW and mutants^*a*^^,^^*b*^

	Mutants	Maximal cleavage activity (μM min^−1^)	Half-maximal activation concn (μM)
The residues in peptide binding pockets	AlgW	165.4 ± 4.46	2.02 ± 0.21
	AlgW-W281A	10.32 ± 1.51	1.51 ± 0.72
	AlgW-L282A	13.24 ± 1.59	2.76 ± 1.19
	AlgW-V284A	63.13 ± 3.82	3.56 ± 0.73
	AlgW-E285A	57.5 ± 4.9	3.84 ± 1.09
	AlgW-K287A	49.42 ± 3.59	6.53 ± 1.38
	AlgW-M342A	NA	NA
	AlgW-R374A	5.45 ± 0.68	ND
The residues involved in domain-domain interactions	AlgW-D149A	8.16 ± 0.85	ND
	AlgW-E151A	34.93 ± 1.87	2.99 ± 0.56
	AlgW-Y212A	39.46 ± 6.17	2.12 ± 0.62
	AlgW-E266A	60.51 ± 5.07	1.08 ± 0.5
	AlgW-R279A	26.87 ± 3.21	2.19 ± 0.99
	AlgW-R347A	63.5 ± 8.42	1.85 ± 0.99

a Apparent activation parameters were determined using the formula of Michaelis-Menten [*Y* = *Vmax* · *X*/(*K_m_* + X)]; the parameters listed are the means of three independent determinations.

b NA, no detectable activity; ND, not determined.

Unlike DegS, whose PDZ is not required for protease catalytic triad restructuring (26, 29), the PDZ domain in AlgW was indispensable for activity (6) (Fig. S1), implying different roles of PDZ in AlgW and DegS. As shown in Fig. 3D, L_3_ and L are located closer to the β6 to β7 turn (where the active residue D153 is located) in AlgW than to that in DegS; AlgW requires interdomain interactions such as R374-E151 and R279-D149 to stabilize the β6 to β7 turn conformation (Fig. 4C and D). It should be noted that R279 and D149 are highly conserved among AlgW/DegS homologues, but the R374-E151 pair is only observed in the AlgW-like group and is substituted by a hydrophobic contact (Y351 and L124 in E. coli DegS) (Fig. 4D and E). R186 of DegS is replaced by Y212 in AlgW to stabilize the interdomain contacts, but Y212 could form extrahydrophobic interactions with M342 and strengthen the association (Fig. 4C and D). Another difference between AlgW and DegS could be found at α4-α7 contacts, in which AlgW forms one salt bridge by E266 and R347 while DegS has more residues (K247, K243, D320, E324) to facilitate the charge-charge attraction in this region (Fig. 4D). Based on the structural analysis, we further introduced Ala substitutions on D149, E151, Y212, E266, R279, and R347 (Table 2). All of those mutants showed significantly reduced activation of AlgW without seriously impairing the peptide activator binding (Table 2). These biochemical results are consistent with and support our analyses of the critical residues involved in interdomain association. Therefore, the PDZ-peptide binding of AlgW also relieves the inhibitory effect of PDZ, but slightly different with DegS, it has to cooperate with the interdomain contacts to convert and maintain the catalytic triad into active conformation.

10.1128/mBio.03299-20.1 FIG S1 Michaelis-Menten plots of cleavage rate constants for peptide-activated AlgW and AlgW-ΔPDZ with the concentration of quenched fluorescent substrates (Abz)-VLAG-Pna (100 μM). The appearance of fluorescent product was monitored at 60-s intervals at fluorescence wavelengths of λex = 310 nm and λem = 420 nm. All solid lines are a fit to the equation rate. Download FIG S1, TIF file, 0.9 MB. Copyright © 2021 Li et al. 2021 Li et al. https://creativecommons.org/licenses/by/4.0/ This content is distributed under the terms of the Creative Commons Attribution 4.0 International license.

### The unique extended L_A_ loop provides a switching mechanism to regulate AlgW function.

The L_A_ loop is known to be an essential regulatory element of HtrA family proteins (19, 30). Because of the high flexibility, our AlgW-tripeptide and AlgWS227A-tripeptide structures, as well as many HtrA protease structures, lack the L_A_ loops (30). Fortunately, L_A_ loops could be reliably modeled in AlgW-decapeptide and AlgWS227A-decapeptide structures, allowing us to explore the conformational regulation mode of the L_A_ loop (Fig. 3D). Sequence alignment reveals that the L_A_ loop of the AlgW-like group is much longer than that of the DegS-like group (Fig. 4E). The AlgW mutation with the deletion of the L_A_ loop (regions 76 to 97) totally lost the protease activity (Table 3), which verified the importance of the L_A_ loop in AlgW function. However, when we replaced this L_A_ loop with the corresponding region (63 to 78) of DegS, it only restored nearly 80% of the catalytic efficiency (apparent *K*_cat_/*K_m_* value calculated from Table 3), demonstrating the distinct regulation effects of the L_A_ loop in different HtrA proteins.

**TABLE 3:** Apparent Michaelis-Menten parameters of AlgW and mutants^*a*^^,^^*b*^

Mutants	V_max_ (μM min^−1^)	K_M_ (μM)	*K*_cat_/*K_m_* (μM^−1^ min^−1^)
AlgW	181.9 ± 5.48	46.44 ± 4.35	58.75 ± 1.77
AlgW-ΔL_A_ (76–97)	NA	NA	NA
AlgW-DegS-L_A_ (63–78)	38.59 ± 2.39	11.99 ± 2.85	48.27 ± 2.99
AlgW-DegS-L_A_ (63–78)-ΔPDZ	NA	NA	NA
AlgW-T71A	154.4 ± 6.43	48.29 ± 6.19	47.96 ± 1.99
AlgW-T72A	766.2 ± 54.15	86.69 ± 15.8	132.58 ± 9.37
AlgW-K73A	187.8 ± 39.36	247.9 ± 93.04	11.36 ± 2.38
AlgW-L106A	NA	NA	NA
AlgW-TTK (71–73)A	1429 ± 99.69	125.1± 19.87	171.34 ± 11.95
AlgW-ESS (103–105)A	873 ± 60.23	123.1 ± 19.44	106.38 ± 7.34

a Apparent Michaelis-Menten parameters were determined using the formula of Michaelis-Menten [*Y* = *V*max · *X*/(*K_m_* + X)]; the parameters listed are the means of three independent determinations. *K*_cat_ for the AlgW trimer was calculated by *K*_cat_ = *V*_max_/(*E*_total_) and (*E*_total_) = 200/3 nM trimers. All solid lines are a fit to the equation rate. Error bars represent standard deviation.

b NA, no detectable activity.

**FIG 5:**
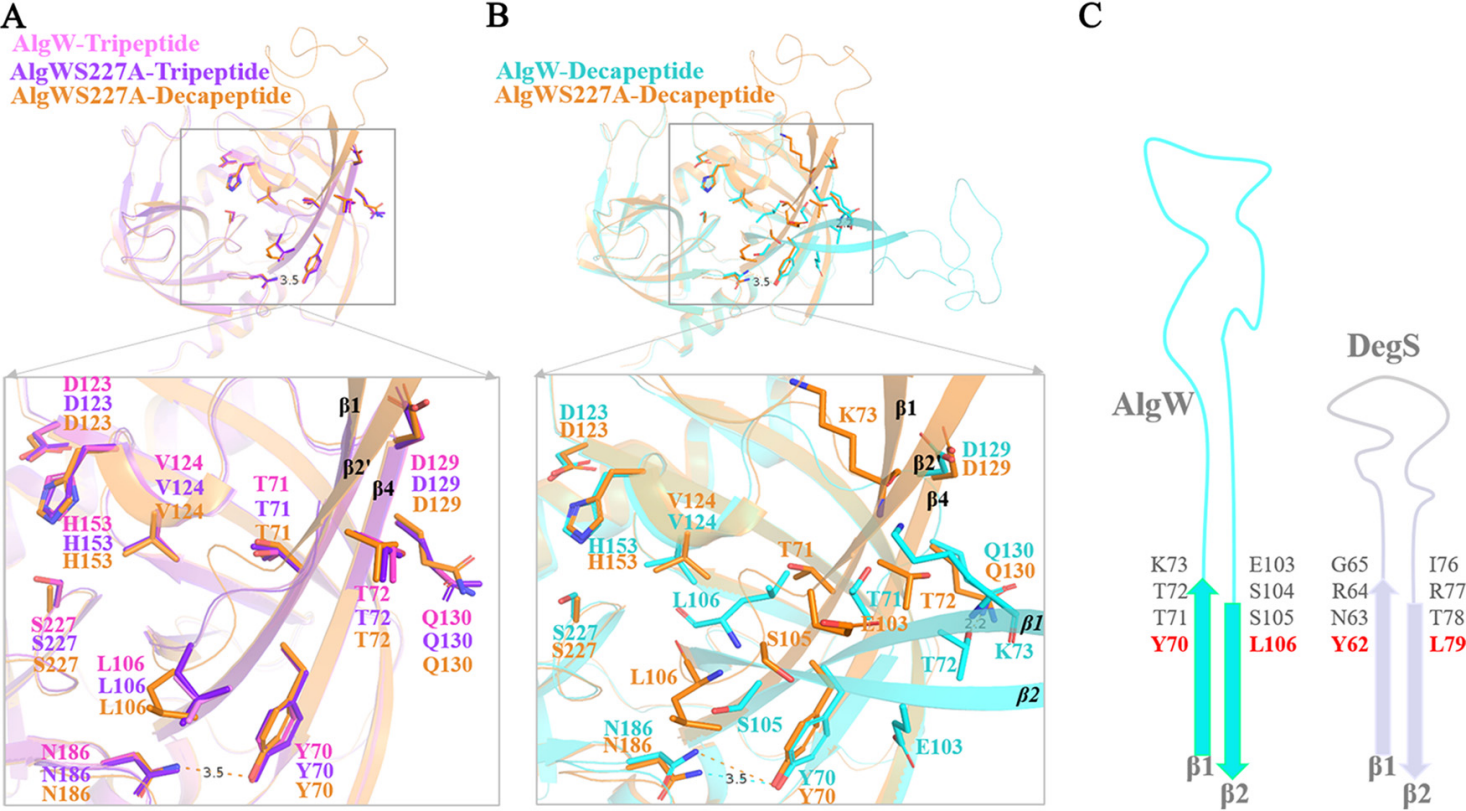
Conformational transition and activity regulation mediated by the L_A_ loop in AlgW. (A and B) Conformational movement of the L_A_ loop in peptide-bound AlgW structures; the key residues involved in the conformational transition of the L_A_ loop are displayed as sticks. AlgWS227A-decapeptide- and tripeptide-bound structures adopted a uniform conformation, which represents the major state of the L_A_ loop. (C) The residue composition of the L_A_ loop hinge region in AlgW and DegS.

By comparing the two sets of decapeptide-bound structures, we found that the L_A_ loop shows joint angle shifts of about 7.65 degrees (Fig. 3D). It seems that the L_A_ loop in the AlgWS227A-decapeptide structure represents the major state of L_A_, because similar to the L_A_ loops in tripeptide-bound structures, they all need to stabilize the antiparallel β1-β4 sheet by forming main-chain hydrogen between D129-Q130 and T72 and maintain the orientation of β1 through the weak interaction between T71 and V124 (Fig. 5A). In the AlgW-decapeptide structure, T72 forms side chain hydrogen interactions with D130 to maintain the β1-β4 association, while in β2, the side chain flipping of L106 not only disrupted T71-V124 interaction but also changed the dihedral angles of S105, leading β2 to form a long antiparallel sheet with β1. Additionally, K73 and E103 also altered their side chain orientations to adapt to the conformational changes (Fig. 5B). Eventually, these structural variations released the L_A_ loop outward and exposed the active center.

According to the above-described analysis, the whole L_A_ loop movement would affect the active site architecture, and residues connecting the L_A_ loop and neighbor β sheets are critical for this structural regulation. However, except for Y70 and L106 (corresponding to Y62 and L79, respectively, in DegS), the rest of the residues are not conserved between AlgW and DegS (Fig. 5C). Single-site or multiple-site mutations on T71-K73 and E103-S105 all increased the *V*_max_ but also the *K_m_* values, especially the T72A and the triple-site mutations, which exhibited 4- to 8-fold enhanced *V*_max_ (Table 3). These observations suggest that the above-described alanine substitutions break the structural constraints and allow the L_A_ loop more flexibility in the conformation transition. Therefore, we may conclude that the distinctive L_A_ motion associates with its different residue compositions among HtrA proteases; meanwhile, the switching between the alternative conformations of L_A_ loop is a key mechanism in AlgW activity regulation.

### Versatile regulation of AlgW is responsible for the efficient P. aeruginosa mucoid conversion.

**FIG 6:**
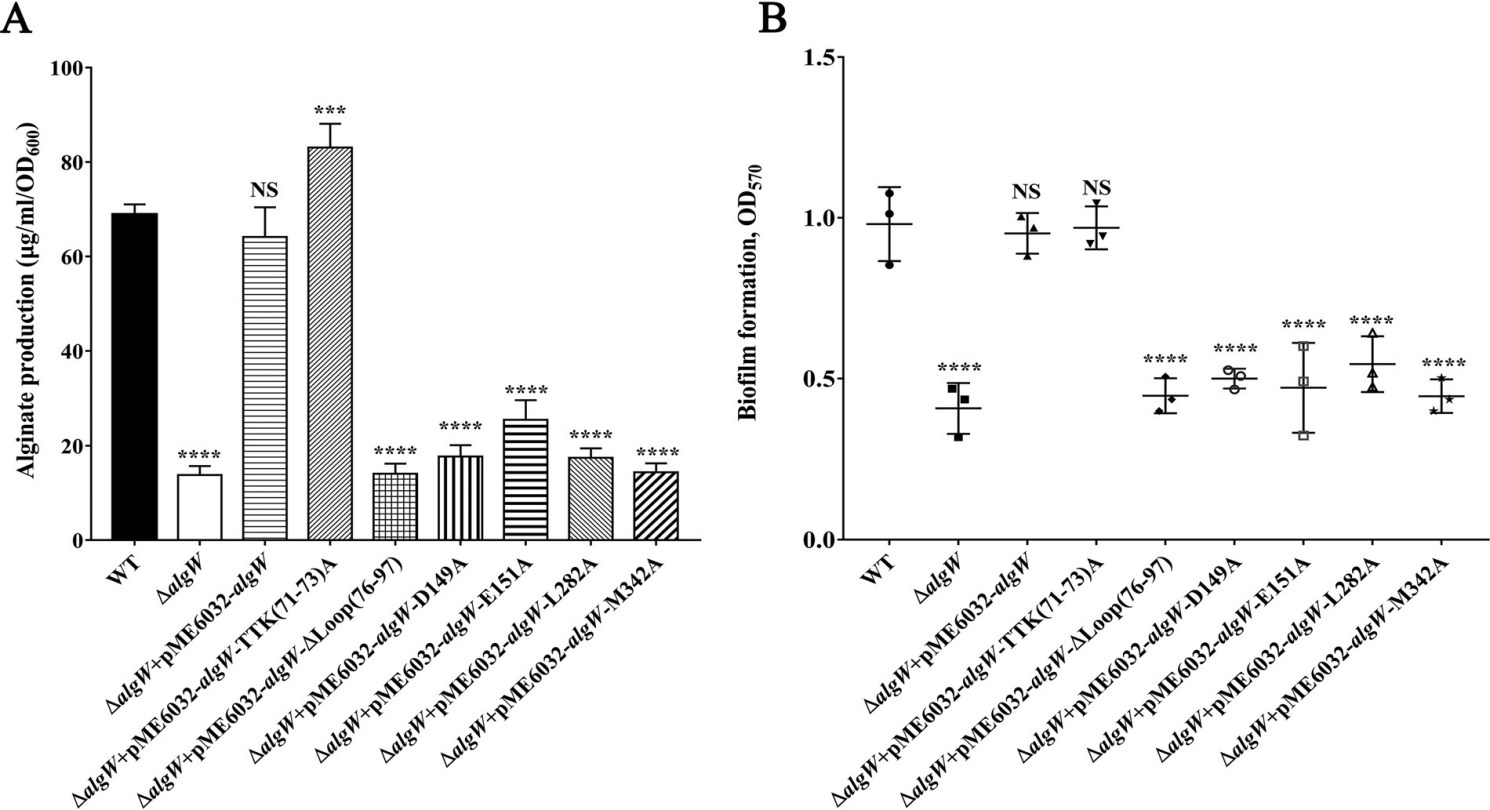
Effect of *algW* knockout and mutants on alginate production and biofilm formation. (A) Alginate production of the Δ*algW*; complementation transformed with plasmid pME6032-*algW* and variants strain compared with that of the wild-type PAO1. Alginate was measured, and the amount of uronic acid in comparison with a standard curve made with d-mannuronic acid lactone was determined. Each bar represents the mean of three independent measurements (± standard error of the mean [SEM]). (B) Biofilm formation of the Δ*algW* and a Δ*algW*+pME6032-*algW* complementation strain compared with that of the wild-type PAO1. Quantification of biofilm biomass via crystal-violet staining and A_570_ was measured using a microplate reader. Data are shown as the change relative to PAO1 and represent three independent experiments. A one-way ANOVA statistical test with equal variances was conducted. The following results were considered significant: *, *P < *0.05; **, *P < *0.01; ***, *P < *0.001; ****, *P < *0.0001.

The essential roles of AlgW in P. aeruginosa alginate synthesis and biofilm formation have been implicated in several reports (4, 11). In order to assess the biological functions of the unique features of AlgW on P. aeruginosa, three groups of AlgW mutations corresponding to peptide activator binding groove (AlgW-L282A, AlgW-M342A), domain-domain interaction sites (AlgW-D149A, AlgW-E151A), specific L_A_ sites [AlgW-ΔL_A_(76-97), AlgW-TTK(71-73)A] were recombined into plasmid pME6032 and supplemented into PAO1-Δ*algW* strain. Then we determined the biochemical indexes of the PAO1-Δ*algW* strain and mutation strains; the results showed that almost all the mutations [except for the mutation of Δ*algW*+pME6032-*algW*-TTK(71-73)A] led to a 2.7- to 4.8-fold decrease of alginate secretion and a 1.7- to 2.2-fold reduced biofilm formation (Fig. 6). Those data verified the regulatory role of key sites of *algW* in physiological processes. It is worth noting that the AlgW-TTK(71-73)A supplement strain showed a 1.2-fold increase in alginate secretion, which was consistent with our structural findings (Table 3) that the L_A_ loop mobility is critical for AlgW activity regulation.

To sum up, the results of mutagenesis and functional studies were consistent with the biological data, which provides a significant proof-effort for explaining the important function of AlgW protease in the diversity of envelope stress responses and physiological regulation.

## DISCUSSION

### Cooperative activation of AlgW by peptide and lipid effectors.

Off-pathway OMPs and LPS in periplasm are implicated in the initiation of the RIP system in Gram-negative bacteria (9). Previous studies have revealed the activation effect of OMP C termini on HtrA-type S1P and the antagonizing mechanism of LPS on heterodimeric anti-sigma factor complex (13). In this article, we biochemically demonstrated that P. aeruginosa S1P AlgW could be activated by either peptide or lipid (Fig. 1). The two distinctive activators showed additive effects to each other, suggesting at least two different mechanisms may be involved in this action (Fig. 1 and 2). Since the lipid-A activation was not observed in E. coli DegS, the dual molecular activation of AlgW represents a novel regulation mechanism for specific HtrA-type proteases. The synergistic binding between peptides and lipids has been found in many PDZ-containing proteins, and the protein-lipid interaction exerts functions such as localization, signaling, and regulation of activity (31–33). Although there is no consensus lipid-binding site on PDZ-containing proteins, previous studies have suggested that the PDZ domain was the dual-effector-binding module and that the lipid-binding site was usually located close to the peptide-binding groove, providing a structural basis for the potential cross-talks between effectors (8, 34, 35). AlgW is the first example of lipid-interacting HtrA protein found in the bacterial RIP pathway; this finding expands our understanding of the diverse enzymatic and regulation characteristics of S1P protease. Our structural and biochemical data (Fig. 3 to 5) provide a rational basis for future studies exploring the detailed mechanism and functions of synergistic activation of HtrA protease by different signals.

### Variation in peptide activator length accounts for the alternative activation states.

The HtrA family protease catalyzes the rate-limiting step in σ^ECF^ activation in response to envelope-stress; OMP-peptide binding is the common mechanism for S1P activation (6). However, despite the variations between AlgW and DegS on the recognition of the C-terminal tripeptide motif in OMPs, AlgW possesses additional sites for anchoring charged residues preceding the hydrophobic tail (Fig. 4). Intriguingly, AlgW showed a more pronounced activation effect when binding to a longer peptide activator; structure data also indicated that decapeptide-bound AlgW was relatively more stable than the tripeptide-bound structure (Fig. 3 and 4). This result is consistent with the fact that decapeptide had a higher binding affinity to AlgW than tripeptide (Fig. S2). Mutations on E285 or K287 resulted in an increased half-maximum activation concentration for decapeptide and dramatically reduced catalytic capacity of AlgW (Table 2), supporting that the additional binding sites are specific and essential for high-affinity binding of the long peptide activator.

10.1128/mBio.03299-20.2 FIG S2 Activity assay of agonist peptide-activated AlgW using the saturated quenched fluorescent substrates (Abz)-VLAG-pNA; 100 μM substrate and 50 nM AlgW or mutants was coincubated at various concentrations of activating peptides (WVF or SVRDELRWVF) in a buffer containing 25 mM Tris (pH 7.5) and 150 mM NaCl at 37°C for 1 h. The final assay volume was 100 μl. The appearance of fluorescent product was monitored at 60-s intervals at fluorescence wavelengths of λex = 310 nm and λem = 420 nm. All data points are the means of three independent determinations and are simulated with solid lines through the formula of Michaelis-Menten. Download FIG S2, TIF file, 1.4 MB. Copyright © 2021 Li et al. 2021 Li et al. https://creativecommons.org/licenses/by/4.0/ This content is distributed under the terms of the Creative Commons Attribution 4.0 International license.

Previous studies have revealed that PDZ domain movements intimately regulated the conformational equilibrium of the DegS active site and the PDZ domain orientations associated with distinct activation states (26). Consistently, AlgW-peptide bound structures also demonstrated different interdomain orientations, in which the joint angles of the PDZ relative to the protease domain are AlgW-WVF (25.62°) < AlgWS227A-WVF (25.74°) < AlgW-decapeptide (25.47 to 26.48°; average, 26.13°) < AlgWS227A-decapeptide (26.65°) (Fig. 3F). Thus, by recognizing additional C-terminal residues of the targeting protein, the PDZ domain of AlgW adopts a more open form and maintains a more stable state, generating stronger substrate cleavage activity. This is in accordance with our enzymatic assay in which the tripeptide-bound AlgW is partially activated, while the decapeptide-bound structures reflect the further activation of AlgW. This diverse peptide activation mechanism is a unique property of AlgW which allows it to conditionally adjust the cleavage activity or functional state.

### Different regulatory mechanisms between AlgW and DegS.

PDZ domains are abundant protein-protein interaction modules found in various species; the interactions between the PDZ and protease domain allows HtrA proteases to modulate the enzymatic activity in response to effector binding (32). The structural mechanism of PDZ-mediated activation was via conformational transitions from PDZ to the exercise module (24). Structural studies of DegS have revealed that the loops (L_1_, L_2_, L_3_, L_D_, L_A_) around the active site had to be organized into certain conformations to achieve the protease activity, but the PDZ domain of inactive DegS captured the L_3_ loop in the interdomain space (20). Although abolishing the inhibitory effect of the PDZ domain by releasing the L_3_ loop is the common activation mechanism between DegS and AlgW, cleavage activity would be partially retained in DegS-ΔPDZ but totally lost in AlgW-ΔPDZ (21). This might be due to the different interdomain interaction networks (Fig. 4), in which the corresponding distributions of hydrophobic and hydrophilic contact pairs are almost reversed in two homologues (Fig. 4D). These variations enforce the dependence of the L_3_ loop of AlgW on the PDZ domain to maintain the active-state conformation after activation. This speculation is consistent with the analysis of M342A and R374A mutants (Table 2), in which the alanine substitutions disrupted the interactions between the PDZ domain and L_3_ loop and hence abolished AlgW activity.

The L_A_ loop is another important regulatory element in HtrA family proteases. L_A_ loops have multiple functions, such as maintaining HtrA oligomeric structure, mediating intra- and intersubunit contacts, keeping the L_1_ and L_2_ loops in a catalytically incompetent conformation, and gating the substrate cavity (19). Our structural data demonstrated the details of how the movement of the L_A_ loop reconstructed the local conformation of the hinge region and directly regulated the accessibility of the active center (Fig. 5). Sequence alignment shows great variability of length and residue content of L_A_ loops between AlgW and DegS groups (Fig. 4E); it might lead to different L_A_ loop mobility and account for the different cleavage efficiencies of the two S1P proteases. In addition, mutagenesis analysis suggested that the increased L_A_ loop flexibility could accelerate the local conformation transition and thus largely improve the cleavage rate, but meanwhile, the more frequent domain switching would impact the substrate binding (Table 3). These results also support the notion that the flexibility of the L_A_ loop is associated with the HtrA protein functionality (19).

### Conclusion.

AlgW is a key protease in the RIP pathway of P. aeruginosa, and its protease activity determines the mucoid conversion and the expression of alginate secretion-related gene clusters (4). It is worth noting that the *in vitro* catalytic efficiency of AlgW is much higher than that of DegS (Fig. 1), indicating that the initiation of the RIP system in P. aeruginosa is faster than that of E. coli. The more sensitive and quicker response of the RIP module enables P. aeruginosa to rapidly boosts its alginate synthesis. On the other hand, accumulations of mis-localized or unfolded proteins and LPS in periplasm are now recognized as critical signals required for the cascade proteolysis process in that RIP pathway (13). In this article, we identified the dual-signal activation in AlgW and demonstrated the synergistic effects between peptide and lipid effectors (Fig. 1 and 2). Furthermore, AlgW could target both MucA and MucB, suggesting an intensive proteolysis process on anti-sigma factors (Fig. 1 and 2). The uncovered unique structural features of AlgW are responsible for its specific biologic functions, such as alginate secretion and biofilm formation (Fig. 6). These structural and functional analysis revealed the structural basis for the distinct activation of AlgW; these new insights into the versatile regulatory mechanism of AlgW confer the strong adaptability and diverse capacities of P. aeruginosa in response to extracellular stresses. Collectively, our study offers a unique opportunity to advance our understanding of the envelope stress-sensing mechanism in bacteria.

## MATERIALS AND METHODS

### Molecular cloning.

Genes encoding AlgW_30-389_ for activity determination and AlgW_52-389_ for crystallization were amplified from the P. aeruginosa genomic DNA by PCR using gene-specific primers (Table S4). These genes were cloned into the pET22b-(LEVLFQ↓GP)-6×His vector using a ClonExpress II one-step cloning kit (Vazyme). The vector pET22b-(LEVLFQ↓GP)-6×His encodes a PreScission protease cleavage site that allows the removal of His-tag. The Blunting Kination Ligation (BKL) kit (TaKaRa) was selected to construct the AlgW mutations.

10.1128/mBio.03299-20.6 TABLE S4 Constructs, oligonucleotides and sequences for AlgW and mutants. Download Table S4, DOCX file, 0.02 MB. Copyright © 2021 Li et al. 2021 Li et al. https://creativecommons.org/licenses/by/4.0/ This content is distributed under the terms of the Creative Commons Attribution 4.0 International license.

### Protein expression and purification.

E. coli BL21(DE3) cells containing expression plasmid vector [pET22b-AlgW-(LEVLFQ↓GP)-6×His] were cultured in Luria-Bertani (LB) medium supplemented with 100 μg/ml ampicillin at 37°C. When the optical density at 600 nm (OD_600_) reached 0.8 to 1.0, protein expression was induced with 0.5 mM isopropyl-β-d-thiogalactoside (IPTG) at 18°C for 15 h.

Bacteria were collected by centrifugation at 4,000 × *g* for 15 min and resuspended in lysis buffer consisting of 25 mM Tris-HCl (pH 7.5), 150 mM NaCl, and 5% glycerol. After sonication, the supernatant was obtained by centrifugation at 15,000 × *g* for 30 min and then coincubated with 4 ml Ni-NTA resin for 1 h. The mixture was washed with lysis buffer complemented with 25 mM imidazole, and target protein was eluted with lysis buffer containing 300 mM imidazole. The protein was further purified with size exclusion chromatography Superdex-75 (GE Healthcare), which was preequilibrated with solution buffer consisting of 25 mM Tris-HCl (pH 7.5) and 150 mM NaCl. Peak fractions were determined by SDS-PAGE analysis. Mutant proteins were expressed and purified as described for the wild type.

### Crystallization, data collection, and structural determination.

Crystallization screens were carried out by mixing protein complex with reservoir buffer at 18°C using the hanging-drop vapor diffusion method. Crystals were obtained in the solution containing index G1 (0.2 M NaCl,0.1 M Tris pH 8.5, 25% [wt/vol] PEG3350) for AlgW-tripeptide, both index D9 (25% PEG3350, 0.1 M Tris pH 8.5) and Proplex G8 (0.8 M sodium/potassium phosphate) for AlgWS227A-tripeptide, PEG(Rx) G2 (2% Tacsimate PH 7.0, 5% 2-propanol, 0.1 M imidazole pH 7.0, 8% PEG3350) for AlgW-decapeptide, and Wizard1&2 A5 (30% PEG400, 100 mM CAPS/sodium hydrochloric pH 10.5) for AlgWS227A-decapeptide. Crystals were soaked in cryo-protectant (reservoir solution supplemented with 20% glycerol) and flash-cooled in liquid nitrogen. Diffraction data were collected on beamline BL18U/BL19U of the Shanghai Synchrotron Radiation Facility (SSRF), China. All diffraction images were integrated, scaled, and merged with the HKL2000 program package (36). The structure of AlgW-WVF was determined by molecular replacement using the PHENIX (37) package with DegS (PDB code: 1SOZ) as a template. The structures of AlgWS227A-tripeptide, AlgW-decapeptide, and AlgWS227A-decapeptide were determined by molecular replacement in Phaser (38) using AlgW-WVF as a search model. All structures were refined in PHENIX in combination with manual building in Coot (39). The final refinement statistics for these structures are summarized in Table 1.

### Muc- and Rse- protease degradation assay.

The Muc- and Rse- protease degradation systems contain equal concentrations of proteases (25 μM AlgW/DegS), substrates (125 μM MucA^peri^/RseA^peri^, 130 μM MucB/RseB), agonist peptides (100 μM tripeptide WVF/YVF or decapeptide SVRDELRWVF/DNRDGNVYYF), and other reagents (100 μM lipid-A or 400 μM DDM). The Muc- cleavage assay was placed in 25 mM Tris-HCl pH 7.5, 150 mM NaCl, and 10% glycerol and reacted at 37°C (9), while the Rse- protease degradation assay was performed at 37°C in a phosphate buffer containing 50 mM NaHPO_4_ (pH 7.5), 200 mM NaCl, 10% glycerol, and 4 mM EDTA (40).

### Enzyme activity assays.

For the enzyme mechanism of AlgW (30–389) and mutants, two internally quenched fluorescent peptide substrates incorporating the peptidase cleavage site of MucA/MucB, Abz-VLAG-pNA and Abz-TVAW-pNA, were designed and synthesized (GL Biochem Shanghai Ltd., China). In the intact peptide, the fluorescence of the anthraniloyl group is quenched by the P-nitroaniline (Abz is a fluorogenic group, and pNA is a quencher of fluorescence). This quenched fluorescence is liberated upon cleavage of peptide by 200 nM AlgW and mutants (200/3 nM for trimer), resulting in increased fluorescence that can then be monitored fluorometrically. Prior to the assay, all enzyme preparations were incubated with 10 mM agonist peptides (WVF or SVRDELRWVF) for 30 min at room temperature. All assays were placed in a buffer containing 25 mM Tris (pH 7.5) and 150 mM NaCl at 37°C for 1 h, and the final assay volume was 100 μl. The fluorescent degradation products were monitored at 60-s intervals at fluorescence wavelength—λex = 310 nm, λem = 420 nm (41). All data points were representative of three independent determinations and were simulated with solid lines with the formula *Y* = *V*_max_ · *X*^h/(*K_m_* ^h + *X*^h) or *Y* = *V*_max_ · *X*^h/(*K*_half_^h + *X*^h) or the Michaelis-Menten formula (Y = *V*_max_ · *X*/[*K_m_* + *X*]). Substrate versus velocity was used to determine *K_m_* and *V*_max_, and agonists versus velocity was used to determine the half-maximal activation concentration and maximum activation rate of agonists. The fitting and calculation of data were carried out using Prism 7 (GraphPad, CA, USA).

The concentration gradients of fluorescent peptide substrates, agonist peptides, and DDM in the determination of the AlgW enzyme kinetic parameters were fluorescent peptide substrate concentration gradients of 3.125 μM, 6.25 μM, 12.5 μM, 25 μM, 50 μM, 100 μM, 200 μM, and 300 μM, agonist peptide concentration gradients of 0.3125 mM, 0.625 mM, 1.25 mM, 2.5 mM, 5 mM, 10 mM, 20 mM, and 40 mM and DDM concentration gradients of 0.0165 mM, 0.03125 mM, 0.0625 mM, 0.125 mM, 0.25 mM, 0.5 mM, 1 mM, and 2 mM.

### Construction of P. aeruginosa *algW* gene deletion and mutation strains.

The deletion of *algW* in P. aeruginosa was constructed according to our previous study (13). The upstream and downstream (600 bp) PCR fragments of *algW* were recombined to the linearized DNA of pEX18Gm with a ligation-free cloning system (5× ligation-free cloning master Mix; abm). The recombinant plasmid was transformed into E. coli S17-1 and then mobilized into P. aeruginosa strain PAO1. Colonies were screened using antibiotic-resistant selection and sucrose-mediated counterselection. The *algW* single-gene deletion strains were further confirmed by PCR and DNA sequencing.

For mutation strains, PCR-amplified *algW* and site-directed mutagenesis were cloned into plasmid pME6032 and transformed into the PAO1-Δ*algW* strain. Finally, the strains were screened using *Pseudomonas* isolation agar (PIA) plates complemented with 200 μg/ml tetracycline.

### Alginate assay.

Alginate was determined as described with our previous research (13). The P. aeruginosa PAO1 and mutant strains were grown on LB medium at 37°C overnight and then subcultured at 1:1,000 into 3 ml fresh LB medium and grown at 37°C for 12 h. The cells were collected and suspended in phosphate-buffered saline (PBS), and the OD_600_ was measured and adjusted to 0.8 by the addition of PBS. The suspensions were analyzed for the amount of uronic acid in comparison with a standard curve made with d-mannuronic acid lactone as previously reported, and the content of alginate in different strains was determined using the sulfuric acid-carbazole colorimetric method.

### Biofilm formation assay.

Biofilm formation was determined as previously described (42). Briefly, overnight bacterial cultures were diluted 100-fold in fresh M63 minimal medium supplemented with magnesium sulfate and arginine. The cell suspension (1 ml) was transferred into each well of a 24-well polyvinyl chloride (PVC) plate (Sigma) and incubated at 37°C. After incubation for 48 h, the M63 minimal medium was removed and the wells were washed twice with a sterilized phosphate-buffered saline (PBS). The cells that adhered to the wells were stained with 0.1% crystal violet for 30 min and then washed twice with PBS. The cell-bound dye was eluted in 2 ml of 95% ethanol, and the absorbance of the eluted solution was measured using a microplate reader at 570 nm. The results are reported for three independent experiments with at least four replicates per experiment.

### Statistics and reproducibility.

All experiments were performed in independent biological triplicate, and the results of replicates were consistent. The fitting and calculation of data were carried out with Prism 7 (GraphPad, CA, USA). All the enzyme kinetic data points were representative of three independent determinations and were simulated with solid lines through the formula *Y* = *V*_max_ · *X*^h/(*K_m_*^h + *X*^h) or *Y* = *Vmax* · *X*^h/(*K*_half_^h + X^h) or the Michaelis-Menten formula (*Y* = *V*_max_ · *X*/[*K_m_* + *X*]). Substrate versus velocity was used to determine *K_m_* and *V*_max_, and agonists versus velocity was used to determine the half-maximal activation concentration and maximum activation rate of agonists; error bars represent the standard deviation.

One-way analysis of variance (ANOVA) was used for the statistical analysis of experimental data *in vivo*. Details of the number of biological replicates are described in the figure legends and Materials and Methods. Error bars represent the standard deviation. A *P* value of <0.05 means that there is a significant difference; a *P* value of <0.0001 was considered extremely significant, which is indicated with ****.

### Data availability.

All data relevant to this study are supplied in the manuscript and supplementary files or are available from the corresponding author upon request. Atomic coordinates of the refined structures have been deposited in the Protein Data Bank (PDB) (https://www.rcsb.org/). The PDB codes for AlgW-tripeptide, AlgWS227A-tripeptide, AlgW-decapeptide, and AlgWS227A-decapeptide are 7CO2, 7CO3, 7CO5, and 7CO7, respectively.
