# Erratum for Li et al., “Molecular Basis of the Versatile Regulatory Mechanism of HtrA-Type Protease AlgW from Pseudomonas aeruginosa”

**DOI:** 10.1128/mBio.01640-21

**Published:** 2021-07-13

**Authors:** Tao Li, Yingjie Song, Liming Luo, Ninglin Zhao, Lihui He, Mei Kang, Changcheng Li, Yibo Zhu, Yalin Shen, Chang Zhao, Jing Yang, Qin Huang, Xingyu Mou, Zhiyong Zong, Jinliang Yang, Hong Tang, Yongxing He, Rui Bao

**Affiliations:** a Center of Infectious Diseases, State Key Laboratory of Biotherapy, West China Hospital, Sichuan University and Collaborative Innovation Center, Chengdu, China; b Department of Cancer Biotherapy Center, The Third Affiliated Hospital of Kunming Medical University (Tumor Hospital of Yunnan Province), Kunming, China; c School of Life Sciences, Lanzhou University, Lanzhou, China; d Department of Laboratory Medicine, West China Hospital, Sichuan University, Chengdu, China

## ERRATUM

Volume 12, no. 1, e03299-20, 2021, https://doi.org/10.1128/mBio.03299-20. In our published article, there was an error in the unit of measurement on pages 4, 5, and 15, which was mainly about the dosage of agonist peptides and which should be “μM” rather than “mM.” The specific changes are as follows:

1. In line 6 of paragraph 4 on page 4 of the text, 10 mM needs to be changed to 10 μM.
2. The dosage of agonist peptides in Fig. 2 needs to be changed to “μM.” The correctly labeled Fig. 2 appears below.
3. The 10 mM in lines 4 and 8 of the legend in Fig. 2 needs to be changed to 10 μM.
4. On page 15, the 10 mM in line 8 of the description of the method for enzyme activity analysis needs to be changed to 10 μM, and “agonist peptide concentration gradients of 0.3125 mM, 0.625 mM, 1.25 mM, 2.5 mM, 5 mM, 10 mM, 20 mM, and 40 mM” in line 20 needs to be changed to “0.3125 μM, 0.625 μM, 1.25 μM, 2.5 μM, 5 μM, 10 μM, 20 μM, and 40 μM.”

**FIG 2:**
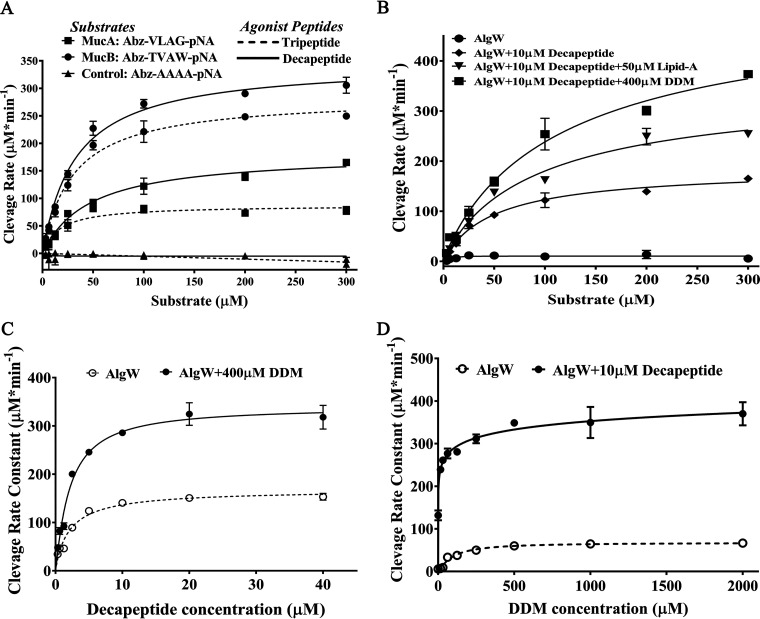
•

