# Supplementary material for: Molecular Basis of the Versatile Regulatory Mechanism of HtrA-Type Protease AlgW from Pseudomonas aeruginosa
Source: mBio. 2021 Feb 23;12(1):e03299-20. doi: 10.1128/mBio.03299-20 (PMC8545111; doi:10.1128/mBio.03299-20)
Supplement: TABLE S1 [file mbio.03299-20-st001.docx]

**Supplementary Table 1. Activity assay of tripeptide or decapeptide activated AlgW.**

| **Substrates *^a^*** | **Agonist peptides *^b^*** | **Vmax (μM min^-1^)** | **Km(μM)** | **Hill constants** |
| --- | --- | --- | --- | --- |
| Abz-VLAG-pNA  (MucA) | Tripeptide | 81.23 ± 4.02 | 14.78 ± 2.16 | 1.51 ± 0.29 |
|  | Decapeptide | 187 ± 12.83 | 50.1 ± 9.65 | 0.95 ± 0.09 |
| Abz-TVAW-pNA  (MucB) | Tripeptide | 264.1 ± 7.31 | 25.05 ± 1.93 | 1.25 ± 0.09 |
|  | Decapeptide | 319.5 ± 6.42 | 26.85 ± 1.48 | 1.26 ± 0.07 |
| Abz-AAAA-pNA  (Control) | Tripeptide | n.a. ***^c^*** | n.a. | n.a. |
|  | Decapeptide | n.a. | n.a. | n.a. |

*^a^* The quenching fluorescence substrate is synthesized according to the determined cleavage sequence of MucA or MucB by AlgW.

***^b^*** the agonist peptides of AlgW are all derived from the C-terminal of periplasmic protein MucE.

***^c^*** n.a., no detectable activity.
