# Supplementary material for: Molecular Basis of the Versatile Regulatory Mechanism of HtrA-Type Protease AlgW from Pseudomonas aeruginosa
Source: mBio. 2021 Feb 23;12(1):e03299-20. doi: 10.1128/mBio.03299-20 (PMC8545111; doi:10.1128/mBio.03299-20)
Supplement: TABLE S2 [file mbio.03299-20-st002.docx]

**Supplementary Table 2. Apparent Michaelis-Menten parameters of AlgW activated by peptides or lipids.**

|  | **Vmax**  **(μM min^-1^)** | **Km**  **(μM)** | **Kcat/Km**  **(min^-1^ μM^-1^)** | **Hill Constants** |
| --- | --- | --- | --- | --- |
| AlgW | n.a. ***^a^*** | n.a. | n.a. | n.a. |
| AlgW+Decapeptide | 187 ± 12.83 | 50.1 ± 9.65 | 55.99 ± 3.84 | 0.95 ± 0.09 |
| AlgW+Decapeptide+Lipid-A | 354.2 ± 43.81 | 95.93 ± 30.25 | 55.38 ± 6.85 | 0.92 ± 0.11 |
| AlgW+Decapeptide+DDM | 523.9 ± 71.57 | 121.6 ± 43.31 | 64.62 ± 8.83 | 0.92 ± 0.1 |

***^a^*** n.a., no detectable activity.
