# Supplementary material for: Molecular Basis of the Versatile Regulatory Mechanism of HtrA-Type Protease AlgW from Pseudomonas aeruginosa
Source: mBio. 2021 Feb 23;12(1):e03299-20. doi: 10.1128/mBio.03299-20 (PMC8545111; doi:10.1128/mBio.03299-20)
Supplement: TABLE S3 [file mbio.03299-20-st003.docx]

**Supplementary Table 3. Apparent activation parameters of AlgW activated by agonist peptides and DDM.**

| **Gradient** | **Groups** | **Maximal Cleavage**  **Activity (μM min^-1^)** | **Half-Maximal**  **Activation concentration (μM)** | **Hill Constants** |
| --- | --- | --- | --- | --- |
| Decapeptide | AlgW | 168.5 ± 8.27 | 2.12 ± 0.33 | 0.94 ± 0.11 |
|  | AlgW+ DDM | 340.2 ± 12.62 | 2.11 ± 0.24 | 1.1 ± 0.11 |
| DDM | AlgW | 67.82 ± 3.02 | 92.02 ± 12.44 | 1.43 ± 0.22 |
|  | AlgW+Decapeptide | 717.8 ± 15.07 | n.d. ***^a^*** | n.d. |

***^a^*** n.d., not determined.
