# Supplementary figures and images for: Molecular Basis of the Versatile Regulatory Mechanism of HtrA-Type Protease AlgW from Pseudomonas aeruginosa
Source: mBio. 2021 Feb 23;12(1):e03299-20. doi: 10.1128/mBio.03299-20 (PMC8545111; doi:10.1128/mBio.03299-20)

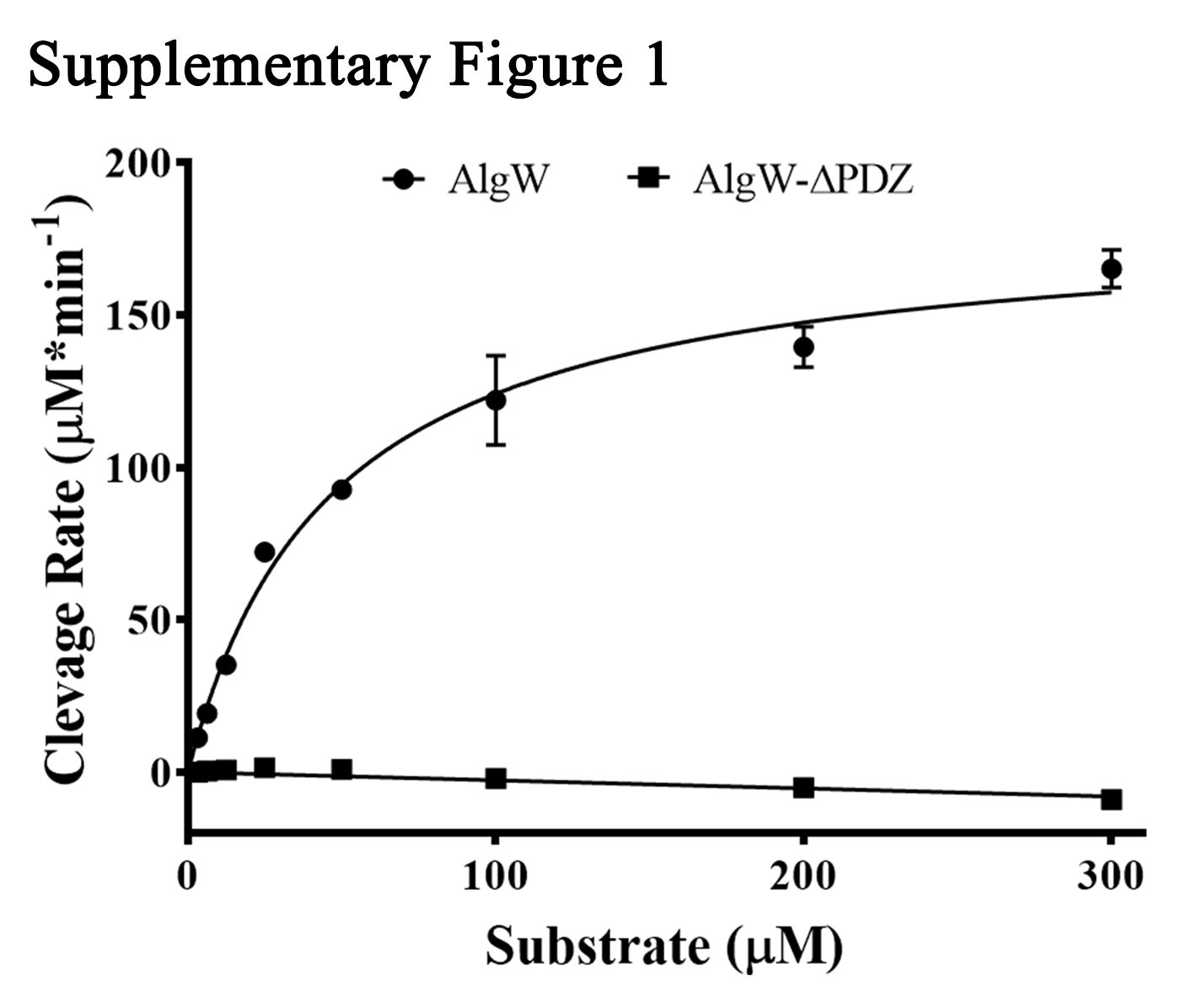

Supplement: FIG S1 [file mbio.03299-20-sf001.tif]

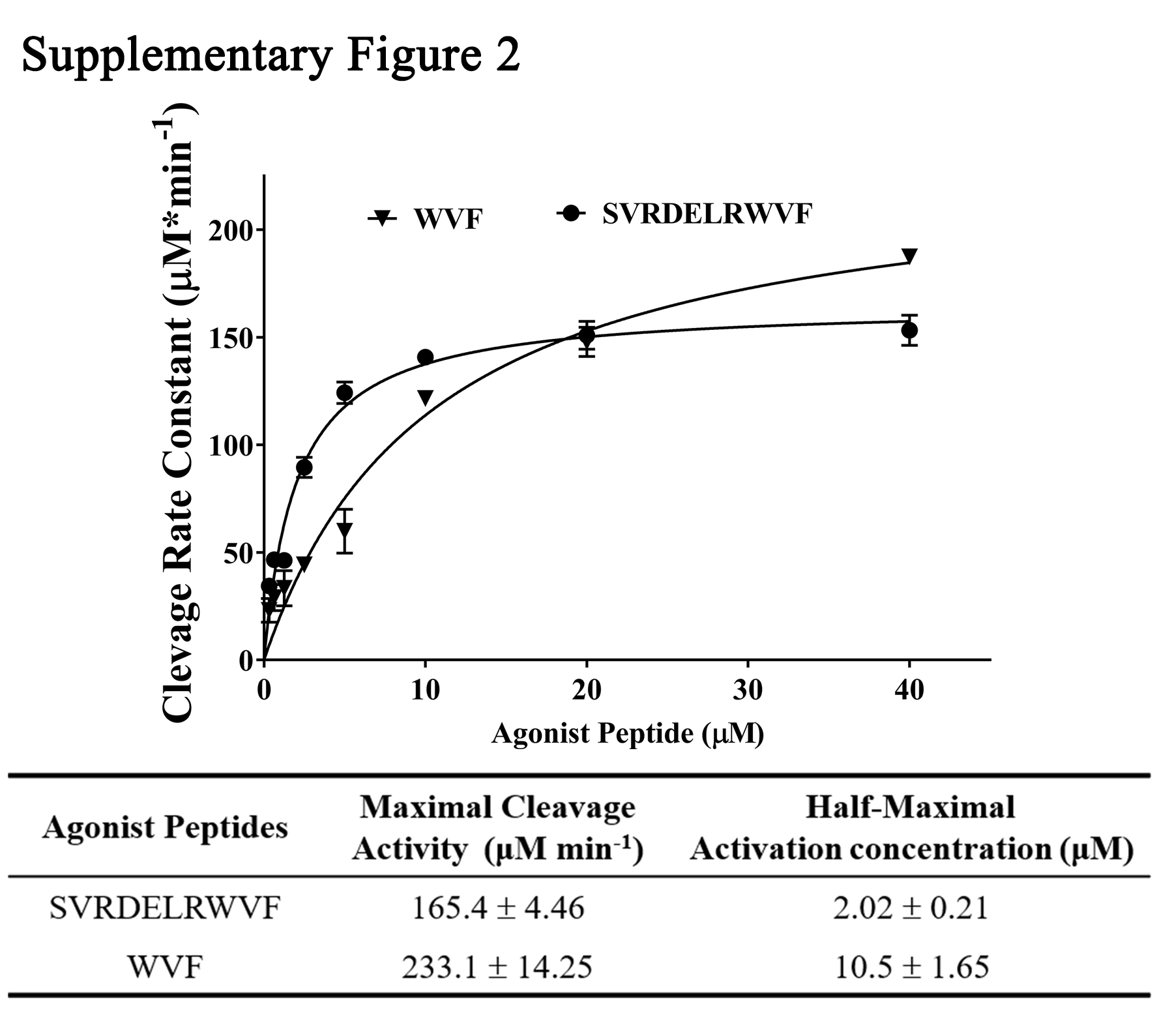

Supplement: FIG S2 [file mbio.03299-20-sf002.tif]
