# Supplementary material for: Molecular Basis of the Versatile Regulatory Mechanism of HtrA-Type Protease AlgW from Pseudomonas aeruginosa
Source: mBio. 2021 Feb 23;12(1):e03299-20. doi: 10.1128/mBio.03299-20 (PMC8545111; doi:10.1128/mBio.03299-20)
Supplement: TABLE S4 [file mbio.03299-20-st004.docx]

**Supplementary Table 4. Constructs, oligos and sequences for AlgW and mutants.**

| **Contructs** | **Oligos and sequences (5’ to 3’)** | |
| --- | --- | --- |
| pET-22b::AlgW(Full Length)-F | 5'TTAAGAAGGAGATATACATATGATGCCCAAGGCCCTGCGTTTCC3' |  |
| pET-22b::AlgW(Full Length)-R | 5'GGTGGTGGTGGTGGTGCTCGAGCTCGCCGCCGTCCTGTTTCTGC3' |  |
| pET-22b::AlgW(30-389)-F | 5'ATGGGCCTGCCACGCCAGGAGGTG3' |  |
| pET-22b::AlgW(30-390)-R | 5'CATATGTATATCTCCTTCTTAAAG3' |  |
| pET-22b::AlgW(50-391)-F | 5'GTGTCCTATGCCAACGCGGTG3' |  |
| pET-22b::AlgW(50-392)-R | 5'CATATGTATATCTCCTTCTTAAAG3' |  |
| pET-22b::AlgW-S227A-F | 5'GGTTGATCGCCGCGTCGGTCTG3' |  |
| pET-22b::AlgW-S227A-R | 5'CCGGCAACGCCGGCGGCGCGCT3' |  |
| pET-22b::AlgW-∆PDZ-F | 5'CTCGAGCACCACCACCACCA3' |  |
| pET-22b::AlgW-∆PDZ-R | 5'GCCGTGCTCGATGATCGACT3' |  |
| pET-22b::AlgW-∆L_A_(76-97)-F | 5'CAACAGAAGCGCATGGAGTCGA3' |  |
| pET-22b::AlgW-∆L_A_(76-97)-R | 5'CTTGCTGACCATCTTGGTGGTG3' |  |
| pET-22b::AlgW+3C-∆HIS-F | 5'TTTCAGGGTCCACTCGAGCACC3' |  |
| pET-22b::AlgW+3C-∆HIS-R | 5'CAGAACTTCGAGCTCGCCGCCG3' |  |
| pET-22b::AlgW-L282A-F | 5'GCCGGCGTCGAGGTCAAGGCGC3' |  |
| pET-22b::AlgW-L282A-R | 5'CCAGCCGCGGATCACCTGGCCG3' |  |
| pET-22b::AlgW-V284A-F | 5'GCCGAGGTCAAGGCGCTGACCC3' |  |
| pET-22b::AlgW-V284A-R | 5'GCCGAGCCAGCCGCGGATCACC3' |  |
| pET-22b::AlgW-E285A-F | 5'GCCGTCAAGGCGCTGACCCCGG3' |  |
| pET-22b::AlgW-E285A-R | 5'GACGCCGAGCCAGCCGCGGATC3' |  |
| pET-22b::AlgW-K287A-F | 5'GCCGCGCTGACCCCGGAACTGG3' |  |
| pET-22b::AlgW-K287A-R | 5'GACCTCGACGCCGAGCCAGCCG3' |  |
| pET-22b::AlgW-M342A-F | 5'GCCAACCAGGTGGCGCGCACCC3' |  |
| pET-22b::AlgW-M342A-R | 5'CGAGCGGCGGCCGTCGCTGGCT3' |  |
| pET-22b::AlgW-Y212A-F | 5'GCCGAAGACTTCATCCAGACCG3' |  |
| pET-22b::AlgW-Y212A-R | 5'GGTGTTCAGGCCGAGCTGGTTG3' |  |
| pET-22b::AlgW-R279A-F | 5'GCCGGCTGGCTCGGCGTCGAGG3' |  |
| pET-22b::AlgW-R279A-R | 5'GATCACCTGGCCGTGCTCGATG3' |  |
| pET-22b::AlgW-E266A-F | 5'GCCGTCATGCAGTCGATCATCG3' |  |
| pET-22b::AlgW-E266A-R | 5'CAGGGCCAGCTTGGTCGGGATG3' |  |
| pET-22b::AlgW-R347A-F | 5'GCCACCCGTCCGGGACAGAAGA3' |  |
| pET-22b::AlgW-R347A-R | 5'CGCCACCTGGTTCATCGAGCGG3' |  |
| pET-22b::AlgW-DegS-L_A_(63-78)-F | 5'AGTTGGTGTTCAAACCACGGTTGTACAGGTTGGCCACTGCCGGAG3' |  |
| pET23b::AlgW-DegS-L_A_(63-78)-R | 5'CTCACAACCAGCTTGAGATCCGCACCCTCGGCTCGGCGGTGATCA3' |  |
| pET-22b::AlgW-W281A-F | 5'GCCCTCGGCGTCGAGGTCAAGG3' |  |
| pET-22b::AlgW-W281A-R | 5'GCCGCGGATCACCTGGCCGTGC3' |  |
| pET-22b::AlgW-R374A-F | 5'GCCCCGCCGCCGGCACCGGCTC3' |  |
| pET-22b::AlgW-V374A-R | 5'CAGGCCGACCTCGGCGGTCAGG3' |  |
| pET-22b::AlgW-D149A-F | 5'GCCCCGGAAACCGACCTGGCCG3' |  |
| pET-22b::AlgW-D149A-R | 5'GCTGCCGACCAACTGGGCGATG3' |  |
| pET-22b::AlgW-E151A-F | 5'GCCACCGACCTGGCCGTGCTGA3' |  |
| pET-22b::AlgW-E151A-R | 5'CGGGTCGCTGCCGACCAACTGG3' |  |
